# The Study of Analgesic Effects of *Leonurus cardiaca* L. in Mice by Formalin, Tail Flick and Hot Plate Tests

**DOI:** 10.1155/2014/687697

**Published:** 2014-09-01

**Authors:** Masoume Rezaee-Asl, Mandana Sabour, Vahid Nikoui, Sattar Ostadhadi, Azam Bakhtiarian

**Affiliations:** ^1^Department of Pharmacology, Pharmaceutical Sciences Branch, Islamic Azad University, Tehran, Iran; ^2^Department of Pharmacology, School of Medicine, Tehran University of Medical Sciences, Tehran 1417613151, Iran

## Abstract

*Leonurus cardiaca*, commonly known as motherwort, is a member of the Lamiaceae family. It has a number of interesting biological activities, for example, sedative and hypotensive, antioxidant, anti-inflammatory, and antimicrobial activities. The aim of the present study was to investigate the effect of alcoholic extract of aerial part of *Leonurus cardiaca* on nociceptive response using formalin, tail flick, and hot plate tests in mice. The acute treatment of mice with an ethanolic extract at doses of 500 and 250 mg/kg by intraperitoneal administration produced a significant antinociceptive in the first and second phases of formalin test, respectively. The hot plate and tail flick tests showed an increase in the antinociceptive effect at dose 500 mg/kg. These results suggest that *Leonurus cardiaca* possesses central and peripheral antinociceptive actions.

## 1. Introduction

Plants have been used for therapeutic applications ever since man has been concerned about his health. For centuries, the world has depended on the useful possessions of plants as a source of medicines [1]. Ethnobotanical investigations done in the last few decades had discovered the analgesic properties of plants mentioned in the traditional information. Numerous herbal preparations are being suggested as analgesic in the traditional information. The exploration for new analgesic compounds from the enormous arrays of medicinal plant resources is growing. This is because such information may hold guarantee for the finding of new therapeutic agents capable of inhibiting, decreasing, or relieving pain [2]. Plants characterize a huge natural supply of valuable compounds that might achieve as lead for the expansion of novel drugs [3]. The exploration of the effectiveness of plant-based drugs used in the traditional medicine has been given great considerations because they are cheap and have little side effects, and, according to World Health Association (WHO), about 80% of the world population still relies chiefly on plant-based drugs [4].

One of these medicinal plant species that has dramatic pharmaceutical values is* Leonurus cardiaca *L. [5].* Leonurus cardiaca*, commonly known as motherwort, is a member of the Lamiaceae family that has been consumed in Asian countries as a traditional remedy against nervous and functional cardiac disorders [6, 7].* Leonurus cardiaca* belongs to the genus* Leonurus* and family Lamiaceae, previously called Labiatae. It is a perennial herb prevalent in Europe, usually found in country areas throughout the plains and hills, as well as in East Asia to the Himalayas and eastern Siberia, Northern Africa, and North America [7]. Motherwort consists of the aerial portions of* Leonurus cardiaca* collected through the flowering time and dried at 35°C and, according to European Pharmacopoeia 7th edition, must encompass a minimum of 0.2% flavonoids expressed as hyperoside [7]. In the aerial parts of* Leonurus cardiac*a, there are ingredients belonging to the group of terpenes, including monoterpenes such as iridoids [8], diterpenes of clerodane [9], furanolabdane, and labdane types [10], triterpenes including ursolic and oleanolic acids [11, 12], nitrogen-containing compounds such as leonurine [13] and stachydrine [14], and phenylpropanoids such as lavandulifolioside [6], as well as flavonoids [15], phenolic acids [16], volatile oils [17], sterols [18], and tannins. Pharmacological reports have established antimicrobial [10, 19], antioxidant [15, 16], and anti-inflammatory [12] effects, as well as the effects of the herb on the heart and circulatory system. Sedative and hypotensive properties have been established in clinical trials. Since pharmacological studies are limited, the use of motherwort is mainly based on traditional suggestions.

Analgesic drugs are one of the most products that are used in numerous diseases for alleviating the pain. Most analgesic drugs, accessible in the market, exhibit an extensive range of adverse effects including gastrointestinal disorders, kidney problems, and other unwanted effects. This situation highlights the need for advent of safe, novel, and effective analgesic compounds. The aim of the present study was to investigate the analgesic activities of aerial part of* Leonurus cardiaca* using three analgesic tests in mice.

## 2. Materials and Methods

### 2.1. Housing and Handling of the Animals

The animals were handled in accordance with the criteria outlined in the Guide for the Care and Use of Laboratory Animals (NIH US publication 86-23 revised 1985). NMRI mice (Pasteur Institute, Tehran, Iran), 6–8 weeks of age, were kept in a controlled environment (22 ± 2°C, 50 ± 5% humidity) under a 12-hour light/dark cycle (light on 08:00–20:00) and had free access to a standard pellet chow and tap water throughout the study. All experiments were conducted at Tehran University of Medical Sciences according to the recommendations of the ethics committee for animal welfare and have been approved by Institutional Animal Care and Utilization Committee.

### 2.2. Preparation of Ethanolic Extract

One hundred grams of aerial parts was placed into a flask; one liter of 96% ethanol was then added. After 24 hours, when the solution had become clear, this was transferred into another flask. One liter of 70% ethanol was then added to the solid residue and after 12 hours the supernatant was again decanted into another flask. Both solutions were then combined and concentrated by vacuum distillation at a temperature of 50°C and 70 rpm rotation speed, until the volume decreased to 1/3 of the initial volume. The solution was then poured into a petri dish and dried in the autoclave at temperatures below 50°C in sterile conditions.

### 2.3. Nociceptive Behavioral Tests

#### 2.3.1. Formalin Test

The formalin test is an acceptable and reliable model of nociception that generates two separate phases of increased licking activity that can be recognized to different nociceptive mechanisms. The initial licking stage lasts for the first five minutes and a late phase takes from 15 to 45 minutes after the injection of formalin. As previously defined, formalin (20 *μ*L of a 2.5% solution) was injected subcutaneously into the dorsal surface of the right hind paw. The animals were then placed under a glass funnel on a glass surface; a mirror was angled at 45 degrees [20]. The pain response time (licking time) was calculated in two periods: 0 to 5 min, the first phase (caused by direct motivation of the nociceptors), and 15 to 45 min, the second phase (inflammatory pain produced by release of inflammatory mediators) [21]. Animals were randomly divided into five groups (*n* = 6). Animals in the negative control group received 0.5 mL of normal saline. Morphine (10 mg/kg, Temad Co., Iran) was injected to animals in the positive control group. The other groups received different doses of* Leonurus cardiaca* extract (125, 250, and 500 mg/kg). All injections were given 30 minutes before the test, intraperitoneally.

#### 2.3.2. Tail Flick Test

The tail flick examination was used to calculate analgesic activity by the method defined by D'amour and Smith 1941 [22], with minor alterations in the procedure. The tail flick method was utilized to study the antinociceptive activity in mice. A radiant heat automatic tail flick analgesiometer was applied to measure reaction latencies. Basal reaction time of animals to radiant heat was recorded by locating the tip (last 1-2 cm) of the tail on radiant heat source. The tail removal from the radiant warmth was taken as end point. The cutoff time of 15 seconds was used to avoid tail injury by heat. Mice were divided into five groups (*n* = 6). Mice were treated with morphine (10 mg/kg), normal saline, and* Leonurus cardiaca* (125, 250, and 500 mg/kg). The latent period of the tail-flick response was determined at 30, 45, 60, 75, and 90 minutes after the administration of drugs.

#### 2.3.3. Hot Plate Test

The hot plate test was used to calculate analgesic activity by the method explained by Eddy and Leimbach [23] with minor modifications. Mice were retained on a hot plate having a stable temperature of 55 ± 1°C. The time taken for either paw licking or jumping was recorded. Each mouse was individually placed on the hot plate in order to find the animal's reaction to electrical heat-induced pain (licking of the forepaws and eventually jumping). The latency until mice showed first signs of discomfort (hind paw lifting, hind paw licking, or jumping) was recorded, before (baseline), and response was determined at 30, 45, 60,75, and 90 min after the administration of normal saline,* Leonurus cardiaca* (125, 250, and 500 mg/kg), and morphine (10 mg/kg).

### 2.4. Data Analysis

Data were analysed using statistical software GraphPad Prism version 5. One-way analysis of variance (ANOVA) test was used to ascertain the significance of variations between the times of licking in formalin test. Two-way repeated measure ANOVA test used to assay the differences in reaction time in tail flick and hot plate tests. Data are shown as mean ± S.E.M. All data were considered significant at *P* < 0.05.

## 3. Results

### 3.1. Formalin Test

**Figure 1:**
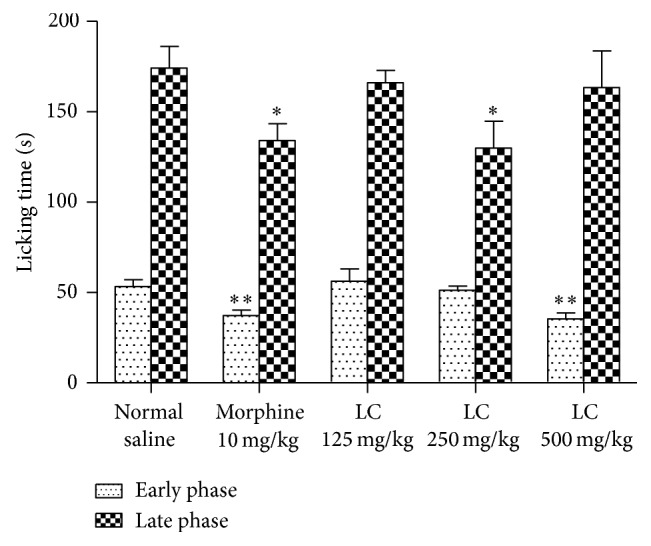
Effects of normal saline, morphine (10 mg/kg), and ethanolic extract of* Leonurus cardiaca* (125, 250, and 500 mg/kg) on the licking time in early and late phases of formalin test. Data are presented as mean ± S.E.M. ∗Significant with late phase of normal saline group (*P* < 0.05). ∗∗Significant with early phase of normal saline group (*P* < 0.01).

Figure 1 shows the results obtained from the formalin test. The treatment of mice with* Leonurus cardiaca* resulted in an inhibition of the formalin-induced licking in the early phase and inflammatory pain (late phase) of the formalin test. In the early phase, the maximum analgesia was observed at dose of 500 mg/kg of* Leonurus cardiaca* extract (*P* < 0.01 compared to control group) and was equal to morphine in dose of 10 mg/kg. In the late phase, the dose of 250 mg/kg of* Leonurus cardiaca* extract showed the most analgesic effects (*P* < 0.05 compared to control group) and it was comparable to morphine in dose of 10 mg/kg.

### 3.2. Tail Flick Test

**Figure 2:**
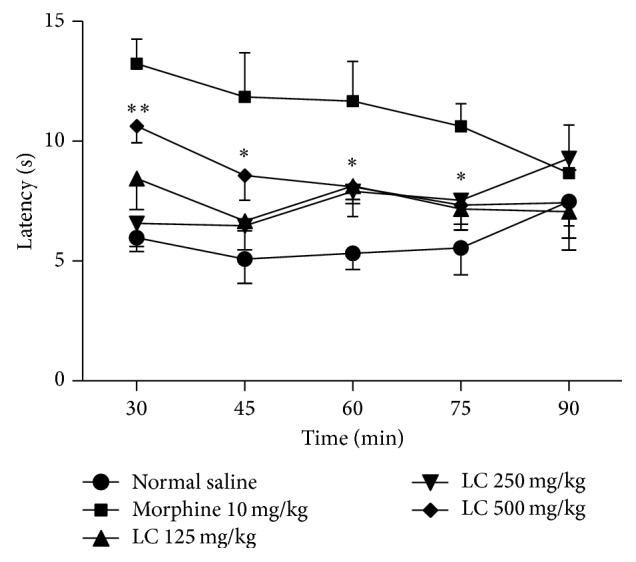
Effects of the ethanolic extract from* Leonurus cardiaca* on the results of the tail flick test in mice. Data are presented as mean ± S.E.M. ∗Significant with correspondence time of normal saline group (*P* < 0.05). ∗∗Significant with correspondence time of normal saline group (*P* < 0.01).

Pretreatment with* Leonurus cardiaca* (250 or 500 mg/kg) demonstrated a significant and dose-dependent antinociceptive activity in the tail flick test (Figure 2). The 500 mg/kg of* Leonurus cardiaca* increased an antinociceptive activity in 30 (*P* < 0.01), 45, 60, and 75 (*P* < 0.01) minutes after injection that were comparable to the normal saline. This effect was significant in time 45 and 60 minutes after injection for doses 125 and 250 mg/kg of* Leonurus cardiaca.* Under similar conditions, treatment with morphine significantly increased latency to thermal stimulation 30 min after administration and the antinociceptive effect was maintained during the entire period of evaluation.

### 3.3. Hot Plate Test

**Figure 3:**
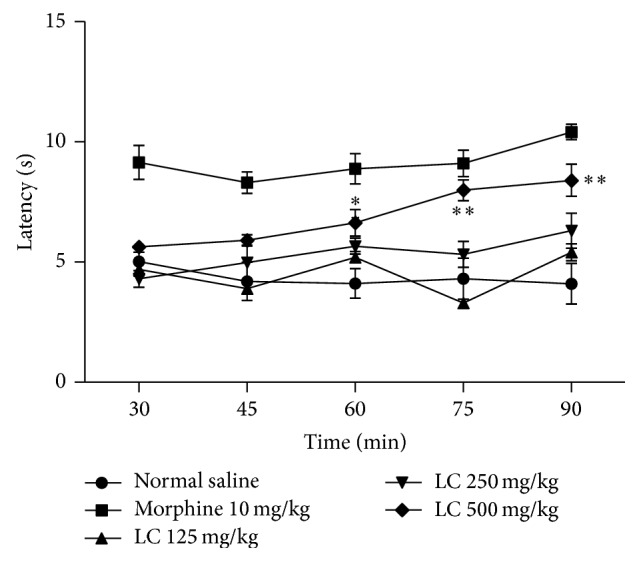
Effects of the ethanolic extract from* Leonurus cardiaca* on the results of the hot plate test in mice. Data are presented as mean ± S.E.M. ∗Significant with correspondence time of normal saline group (*P* < 0.05). ∗∗Significant with correspondence time of normal saline group (*P* < 0.01).

The results in Figure 3 show that the treatment of mice with morphine (10 mg/kg i.p.) increased the latency response in the hot plate test from 30 to 120 minutes after treatment. On the other hand,* Leonurus cardiaca* significantly influence the reaction time of the animals to the hot plate at doses of 500 mg/kg in 45 and 60 minutes (*P* < 0.05) and 75 and 90 minutes (*P* < 0.01) after treatment.

## 4. Discussion

In this study, we pointed to investigate the potential antinociceptive effect of aerial part ethanol extract from* Leonurus cardiaca* by chemical and thermal models of nociception. The first test to assess the antinociceptive property* Leonurus cardiaca* was the formalin test. Formalin is a noxious stimulus frequently used in animal behavioral experimentations. The formalin test initially described by Dubuisson and Dennis [21] and categorized by the first phase, which is induced by direct formalin stimulation of the sensorial C-fibers followed by substance P release [24], and the second phase (inflammatory) principally due to a following inflammation reaction in the peripheral tissue mediated by the release of various inflammatory mediators has been related with the augmented level of PG, stimulation of COX, and release of nitric oxide (NO) [25, 26]. The biphasic nature of pain response in this test, which presents diverse pathological procedures, can be used to clarify the probable mechanism involved in analgesia [27]. Centrally acting drugs, such as opioids, prevent both phases of pain, whereas peripheral-acting drugs such as acetylsalicylic acid, which inhibit COX activity, only inhibit the second phase [24, 28]. Our results showed that the* Leonurus cardiaca* extract at dose of 500 mg/kg and 250 mg/kg was more effective in the first and second phases, respectively, as well as morphine 10 mg/kg, suggesting possible peripheral and central antinociceptive mechanism. The second phase of formalin test is related to a peripheral inflammatory process. This extract was possibly able to inhibit this inflammation, so it can be deduced that peripheral mechanisms might also be involved in antinociceptive effects. It is generally believed that centrally acting analgesics possess effects on both phases, while peripherally acting analgesics affect only the first phase. This is because the injected formalin into the paw results in release of several neurotransmitters including glutamate and aspartate in dorsal horn of spinal cord. So the early phase of the formalin test represents the transmission of nociceptive impulses, while late phase is associated with the development of a delayed inflammatory response.

Opioid analgesics suppress both of early and late phases of formalin test, while none steroidal anti-inflammatory drugs (NSAIDs) mainly act on the late phase. As shown in Figure 1, morphine could alleviate both of phases, whereas* Leonurus cardiaca* at dose of 250 mg/kg diminished only the formalin-induced pain of the late phase. This effect maybe contributed to presence of some anti-inflammatory compounds which may act like NSAIDs.

Demonstrating in Figure 1,* Leonurus cardiaca* extract only at the maximum administrated dose (500 mg/kg) was capable of reducing the formalin-induced pain in the early phase, and other doses (125 and 250 mg/kg) did not show such effect. A pharmacokinetic involvement may partly clarify this finding that describes that* Leonurus cardiaca* extract at the maximum dose (500 mg/kg) could pass through blood-brain barrier and show its analgesic properties in early phase, which is mainly central, while lower doses did not display such effect perhaps due to poor passing via this barrier.

Aiming to study the spinal antinociceptive action, we performed the tail flick test. This model, like the hot plate test [22, 29], measures animal nociceptive response latencies to thermal stimulus but tail flick is principally a spinal response and hot plate is predominantly supraspinal [30–32]. Treating the animals with* Leonurus cardiaca*, at dose 500 mg/kg, alters mouse latency to painful thermal stimulus in tail flick and hot plate tests. These findings suggest that central and peripheral mechanisms are involved in the antinociceptive activity of the extract. Demonstrating in Figure 2,* Leonurus cardiaca* extract only at the maximum dose (500 mg/kg) could alleviate the pain in all times of tail flick test, whereas the lower doses (125 and 250 mg/kg) reduced only late pain (times 60 and 75 minutes). This finding is similar to results of formalin test; in both of them,* Leonurus cardiaca* extract only at dose of 500 mg/kg was capable of diminishing the early pain, which is mainly central, and, as aforementioned, this event can be contributed to pharmacokinetics.

In this study, it was shown that the administration of ethanolic aerial part extract of* Leonurus cardiaca* possesses antinociceptive effects in tail flick, hot plate, and both phases of formalin test.

## 5. Conclusions

The results obtained in this study indicate that the extract possesses analgesic properties, which are mediated through peripheral and central inhibitory mechanisms. This could provide a rationale for the use of this plant in pain and inflammatory disorders in folk medicine. Further studies should be performed for the isolation of new chemical constituents of the plant as well as for a better understanding of the mechanism of antinociceptive activity presented by the extract.
